# Markers of Oxidative Stress during Diabetes Mellitus

**DOI:** 10.1155/2013/378790

**Published:** 2013-12-17

**Authors:** Brahm Kumar Tiwari, Kanti Bhooshan Pandey, A. B. Abidi, Syed Ibrahim Rizvi

**Affiliations:** ^1^Department of Biochemistry & Biochemical Engineering, Sam Higginbotom Institute of Agriculture, Technology & Sciences, Allahabad 211007, India; ^2^Department of Biochemistry, University of Allahabad, Allahabad 211002, India

## Abstract

The prevalence of diabetes mellitus is rising all over the world. Uncontrolled state of hyperglycemia due to defects in insulin secretion/action leads to a variety of complications including peripheral vascular diseases, nephropathy, neuropathy, retinopathy, morbidity, and/or mortality. Large body of evidence suggests major role of reactive oxygen species/oxidative stress in development and progression of diabetic complications. In the present paper, we have discussed the recent researches on the biomarkers of oxidative stress during type 2 diabetes mellitus.

## 1. Introduction

Diabetes mellitus is a group of metabolic diseases characterized by hyperglycemia resulting from defects in insulin secretion and insulin action or both. The chronic hyperglycemia is associated with long-term damage, dysfunction, and failure of normal functioning of various organs, especially the eyes, kidneys, nerves, heart, and blood vessels [1, 2]. Diabetes-specific microvascular disease is a leading cause of blindness, renal failure, and nerve damage [3].

The prevalence of diabetes is rising all over the world due to population growth, aging, urbanisation, and the increase of obesity due to physical inactivity. Unlike the West, where the older are most affected, diabetes in Asian countries is comparatively high in young to middle-aged people. All these complications have long-lasting adverse effects on a nation's health and economy, especially for developing countries. As per estimate of the International Diabetes Federation (IDF), the total number of people in India with diabetes which was around 50.8 million in 2010 would be 87.0 million by 2030 [4].

Hyperglycaemia generates reactive oxygen species (ROS), which in turn cause damage to the cells in many ways. Damage to the cells ultimately results in secondary complications in diabetes mellitus [5, 6]. In the present paper, we have discussed the markers of oxidative stress in diabetes mellitus. The involvement of ROS in the aetiology and the development of late complications have also been addressed. The review further examines the main toxic effects of ROS on lipid, protein, glutathione metabolism, catalase, superoxide dismutases, and antioxidant capacity of plasma.

## 2. Diabetic Complications

Diabetes is a major source of morbidity, mortality, and economic cost to the society. People with diabetes showed the risk of the development of acute metabolic complications such as diabetic ketoacidosis, hyperglycaemic hyperosmolar nonketotic coma, and hypoglycaemia [7, 8]. In addition to this, diabetics are also at risk of experiencing chronic complications such as coronary heart diseases, retinopathy, nephropathy and neuropathy, and foot ulceration [9]. A variety of factors influence the development of diabetic pathologies. Insulin resistance which develops from obesity and physical inactivity acts as substrate for genetic susceptibility [10]. Since food intake influences the amount of insulin required to meet blood glucose target goals, the food especially carbohydrate intake could contribute to the pathology of diabetes. Dietary carbohydrate influences postprandial blood glucose levels the most and is the major determinant of meal-related insulin requirements. The intermediate- or longer-acting insulin usually covers the effects of protein and fat. It has been shown that low carbohydrate ketogenic diet is effective in the amelioration of many of the deleterious consequences of diabetes [11]. It has been observed that insulin secretion declines with advancing age, and this decline may be accelerated by genetic factors. Insulin resistance typically precedes the onset of type 2 diabetes and is commonly accompanied by other cardiovascular risk factors: dyslipidemia, hypertension, and prothrombotic factors [12].

It has been estimated that expenditure of diabetic persons on health is about four folds higher than that of general healthy population. Recent prospective studies have provided unequivocal evidence on crucial role of prolonged hyperglycaemia in the development of chronic diabetic complications [2, 6, 13].

## 3. Role of Oxidative Stress in Diabetes

Oxidative stress plays a pivotal role in cellular injury from hyperglycemia. High glucose level can stimulate free radical production. Weak defence system of the body becomes unable to counteract the enhanced ROS generation and as a result condition of imbalance between ROS and their protection occurs which leads to domination of the condition of oxidative stress [14, 15]. A certain amount of oxidative stress/ROS is necessary for the normal metabolic processes since ROS play various regulatory roles in cells [16]. ROS are produced by neutrophils and macrophages during the process of respiratory burst in order to eliminate antigens [17]. They also serve as stimulating signals of several genes which encode transcription factors, differentiation, and development as well as stimulating cell-cell adhesion, cell signalling, involvement in vasoregulation, fibroblast proliferation, and increased expression of antioxidant enzymes [16, 18, 19]. However over- and/or uncontrolled production of ROS is deleterious. Due to oxidative stress the metabolic abnormalities of diabetes cause mitochondrial superoxide overproduction in endothelial cells of both large and small vessels, as well as in the myocardium [2, 20]. Oxidative stress acts as mediator of insulin resistance and its progression to glucose intolerance and installation of diabetes mellitus, subsequently favouring the appearance of atherosclerotic complications, and contributes to rise in many micro- and macrovascular complications [21].

Hyperglycaemia causes tissue damage through multiple mechanisms including increased flux of glucose and other sugars through the polyol pathway, increased intracellular formation of advanced glycation end products (AGEs), increased expression of the receptor for AGEs and its activating ligands, activation of protein kinase C isoforms, and overactivity of the hexosamine pathway [22]. Atherosclerosis and cardiomyopathy in type 2 diabetes are caused in part by pathway-selective insulin resistance, which increases mitochondrial ROS production from free fatty acids, and by inactivation of antiatherosclerosis enzymes by ROS. Diabetics differ significantly in their sensitivity to ROS. Inflammatory damage that characterizes type 1 diabetes is mediated at least in part through islet ROS, and in type 2 diabetes, the high nutrient flux and consequent ROS production appear to mediate loss of *β*-cell function. In insulin-sensitive tissues including muscle, liver, and heart, high fatty-acid flux leads to oxidative damage, whereas noninsulin-sensitive tissues including the eye, kidney, and nervous system are exposed to both high circulating glucose and fatty acid levels and, consequently, ROS-induced diabetic complications [23].

## 4. Oxidative Stress-Induced Alterations in Diabetes

**Figure 1:**
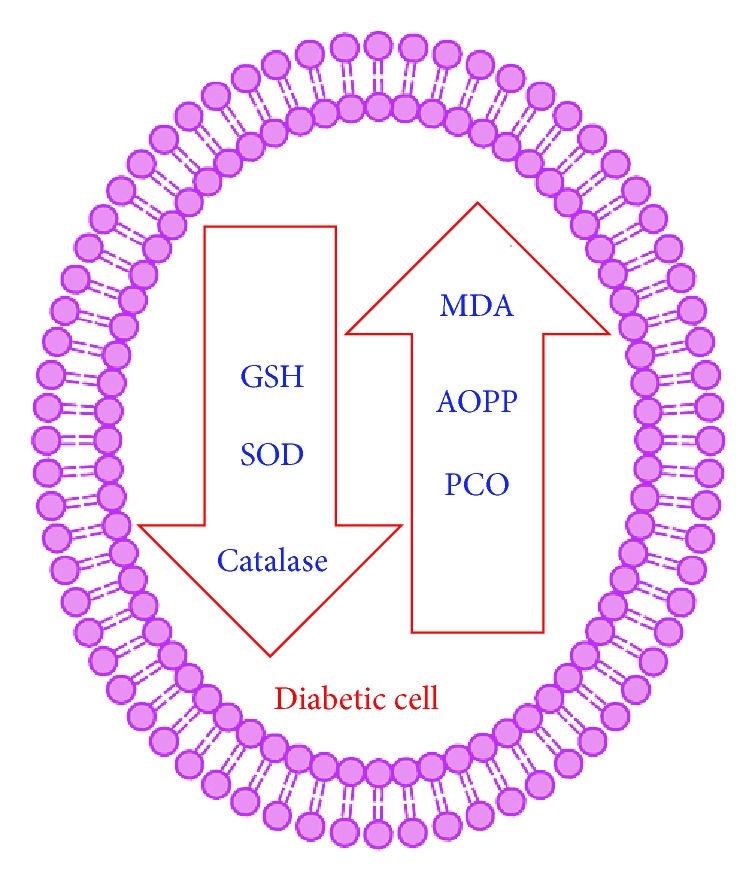
Schematic representation of the status of oxidative stress markers during diabetes. MDA: malondialdehyde, AOPP: Advanced oxidation protein products, PCO: protein carbonyls, GSH: reduced glutathione, and SOD: superoxide dismutase.

Oxidative stress in diabetes mellitus causes several adverse effects on the cellular physiology. This is particularly relevant and dangerous for the islet, which is among those tissues that have the lowest levels of intrinsic antioxidant defences. Multiple biochemical pathways and mechanisms of action have been implicated in the deleterious effects of chronic hyperglycemia and oxidative stress on the function of vascular, retinal, and renal tissues [24–26]. Here we have described the oxidative stress-induced alterations in major biomolecules in the cell and status of plasma antioxidant potential during type 2 diabetes (Figure 1).

### 4.1. Lipid Peroxidation

Lipids are reported as one of the primary targets of ROS. Hydroperoxides have toxic effects on cells both directly and through degradation to highly toxic hydroxyl radicals. They may also react with transition metals like iron or copper to form stable aldehydes, such as malondialdehyde (MDA), that damage cell membranes [27]. Peroxidation of lipids produces highly reactive aldehydes, including MDA, acrolein, 4-hydroxynonenal (HNE), 4-oxononenal (ONE), and isolevuglandins (IsoLGs) [28]. It has been reported that peroxyl radicals can remove hydrogen from lipids, producing hydroperoxides that further propagate the free-radical pathway [29]. MDA has been documented as a primary biomarker of free radical mediated lipid damage and oxidative stress [30].

Significant changes in lipid metabolism and structure have been reported in diabetes, particularly in patients with vascular complications [31]. Increased level of MDA in diabetics suggests that peroxidative injury may be involved in the development of diabetic complications. The increase in lipid peroxidation is also an indication of decline in defence mechanisms of enzymatic and nonenzymatic antioxidants [32]. Oxidized lipids are able to produce MDA as a decomposition product and the mechanism is thought to involve formation of prostaglandins, like endoperoxides, from polyunsaturated fatty acid (PUFA) with two or more double bonds [33]. Increased MDA level in plasma, serum, and many others tissues has been reported in diabetic patients [34, 35]. In 1991, Baynes, followed by Ramesh et al. in 2012 [36, 37], reported that lipid peroxidation in diabetes induced many secondary chronic complications including atherosclerosis and neural disorders. Yang et al. (2009) observed greater serum lipid peroxidation evaluated in terms of MDA in hyperglycemic mice and proposed that the increase in lipid peroxidation exacerbated the occurrence of myocardial infraction through NADPH oxidase activation [38].

Lipid peroxidation of cellular structures is thought to play a key role in atherosclerosis. Significantly higher values of thiobarbituric acid-reactive substances (TBARS) in the red blood cells as well as in serum and decreased erythrocyte antioxidant enzyme activities have been reported in diabetic condition [39, 40]. Increased lipid peroxidation presents a close relationship with the high glycemic levels and oxidative stress in diabetes mellitus [35, 41]. Recently, a clinical study performed by Bandeira and coworkers (2012) aimed at characterizing blood oxidative stress in diabetic patients reported a significant higher lipid peroxidation which showed a close relationship with high glucose levels as observed by the fasting glucose and HbA1c levels [35].

### 4.2. Protein Oxidation

Proteins are the important vital biomolecules of the cell. They are involved in many physiological functions including cell signalling and transport across the cells. Proteins are another potential target of ROS, whose structure and function can be affected by modification. There are many side chain targets for protein oxidation including cysteine, methionine, and tyrosine. Carbonyls are the oxidation product of proteins and are reported as the potent biomarker of oxidative stress [42]. They represent the stable end product generated upon formation of transient radical species, such as chloramines and nitrogen/carbon radicals, which are induced by oxidant stimuli. Glycation has been reported to induce the formation of protein carbonyls, such as ketoamine derivatives, thus generating reactive radicals and perpetuating a vicious cycle [43].

Increased protein carbonyl content has been reported in different cells and plasma of the diabetic patients [15, 42]. Damage of proteins followed by accumulation of their oxidation products affects cellular physiology adversely. Increased glycol- and lipooxidation are reported as one of the major factors in the accumulation of nonfunctional damaged proteins [44].

Gradinaru et al. (2013) have reported the significance of the oxidative and glycoxidative protein damage in subjects with prediabetes and type 2 diabetes mellitus. AGEs, low-density lipoprotein susceptibility to oxidation (oxLDL) and nitric oxide metabolic pathway products (NOx), are documented as important biomarkers for evaluating the association between diabetes and protein status in diabetic patients [45]. AGEs are formed through nonenzymatic amino-carbonyl interactions between reducing sugars or oxidised lipids and proteins, amino phospholipids, or nucleic acids [46]. The generation of AGEs may lead to intracellular modifications of proteins, including those involved in the regulation of gene expression [47]. Many studies on animals as well as on humans have frequently reported the relationship between hyperglycaemia, oxidative stress, and formation of AGEs [47–49]. AGEs are capable of modifying the circulating proteins in the blood that have receptors for AGEs, activating them followed by inducing the production of inflammatory cytokines and growth factors in endothelial cells [50].

Advanced oxidation protein products (AOPPs) are the recently investigated marker of protein oxidation during oxidative stress which represents the overall status of the protein in the cell/tissue [51, 52]. In chronic oxidative stress, AOPPs are formed by reactions between plasma proteins and chlorinated oxidants. Their increased levels are reported during type 2 diabetes. Significant positive association between plasma levels of AOPPs and TBARS during diabetes indicates that proteins are equally targeted by ROS as the lipids [53].

Oxidation of proteins in diabetics affects many physiological functions [54, 55]. Increased protein carbonyls as well as AOPPs level in diabetic patients underlie the importance of the protein conformational changes in the pathogenesis of diabetic nephropathy [56]. AOPPs known as proinflammatory and prooxidative compounds that accumulate in aging patients with diabetes may play a major role in increasing prevalence of endothelial dysfunction and subsequent cardiovascular diseases. AOPPs contain abundant dityrosines which allow crosslinking, disulfide bridges, and carbonyl groups and are mainly formed by chlorinated oxidants, hypochloric acid and chloramines resulting from myeloperoxidase activity [57]. Several studies have pointed out that AOPPs and oxidative stress markers increase in adult subjects with type 2 diabetes with and without micro-/macrovascular complications [45, 54].

### 4.3. Glutathione Level

Glutathione (GSH), a tripeptide, *γ*-L-glutamyl-L-cysteinylglycine, is present in all mammalian tissues at 1–10 mM concentrations (highest concentration in liver) as the most abundant nonprotein thiol that defends against oxidative stress [58]. GSH can maintain SH groups of proteins in a reduced state, participate in amino acid transport, detoxify foreign radicals, act as coenzyme in several enzymatic reactions, and also prevent tissue damage [59]. It is an efficient antioxidant present in almost all living cells and is also considered as a biomarker of redox imbalance at cellular level [60]. There are several reports that claim reduced level of GSH in diabetes [61, 62]. Decreased GSH level may be one of the factors in the oxidative DNA damage in type 2 diabetics [63].

As a consequence of increased oxidative status, GSH showed the frequent alteration in its concentration. Plasma GSH/GSSG showed a significant decrease in type 2 diabetes as compared to normal [62]. Hyperlipidemia, inflammation, and altered antioxidant profiles are the usual complications in diabetes mellitus as results decreased GSH/GSSG ratio [64]. Abnormal GSH status is involved in *β*-cell dysfunction and in the pathogenesis of long-term complications of diabetes. The dysregulation is widely implicated in disease states [65]. Glutathione reductase (GSR) plays an important role through the reduction of GSSG to GSH and oxidation of NADPH to NAD+. GSSG is unable to perform antioxidant functions; however, GSH can be reclaimed from GSSG through the use of glutathione reductase (GSR) by the use of NADPH as a cofactor. Unfortunately, this GSH system can be overwhelmed if ROS are produced in excess [66]. Uncontrolled type 2 diabetes has severely deficient synthesis of GSH attributed to limited precursor availability. Dietary supplementation with GSH precursor amino acids can restore GSH synthesis and lower oxidative stress and oxidant damage in the face of persistent hyperglycemia [67]. It has been observed that GSH deficiency in diabetics increased their susceptibility to melioidosis. It is hypothesized that maintenance of GSH redox state may be a new therapeutic avenue to protect diabetic patients against some intracellular bacterial pathogens [68].

### 4.4. Catalase

Catalase is an antioxidative enzyme present nearly in all living organisms. It plays an important role against oxidative stress-generated complications such as diabetes and cardiovascular diseases [69]. Catalase acts as main regulator of hydrogen peroxide metabolism. Hydrogen peroxide is a highly reactive small molecule formed as natural by-product of energy metabolism. Excessive concentration of hydrogen peroxide may cause significant damages to proteins, DNA, RNA, and lipids [70]. Catalase enzymatically processes hydrogen peroxide into oxygen and water and thus neutralizes it. Increased risk of diabetes has been documented in patients with catalase deficiency. The deficiency of this enzyme leads, in the *β*-cell, to an increase in oxidative stress and ultimately to a failure of this cell type. *β*-cells are rich in mitochondria, and thus this organelle might be a source of ROS [71].

Catalase protects pancreatic *β*-cells from damage by hydrogen peroxide [72]. Low catalase activities, which have been reported in patients with schizophrenia and atherosclerosis [73], are consistent with the hypothesis that long-term oxidative stress may contribute to the development of a variety of late-onset disorders, such as type 2 diabetes [74]. Deficiency of catalase increases mitochondrial ROS and fibronectin expression in response to free fatty acids, which were effectively restored by catalase overexpression or N-acetyl cysteine [75]. Low catalase activities can cause methemoglobinaemia and hemolytic anemia which may be attributed either to deficiency of glucose-6-phosphate dehydrogenase or to other unknown circumstances and also may damage heme proteins, cause cell death, and, together with redox active metal ions, produce highly toxic hydroxyl radicals [76, 77].

Patel and coworkers [78], during investigation of hyperglycemia-induced functional changes: superoxide, hydrogen peroxide production, mitochondrial membrane polarization, and gene expression fingerprints of related enzymes in endothelial cells, have reported that hyperglycemia increased hydrogen peroxide production, hyperpolarized mitochondrial membrane, and downregulated CAT gene expression.

### 4.5. Superoxide Dismutase

Superoxide dismutase (SOD) is the antioxidant enzyme that catalyses the dismutation of superoxide anion (O_2_^−^) into hydrogen peroxide and molecular oxygen [79, 80]. SOD plays important protective roles against cellular and histological damages that are produced by ROS. It facilitates the conversion of superoxide radicals into hydrogen peroxide, and in the presence of other enzymes it converted into oxygen and water [81]. All mammalian tissues contain three forms of SOD: Cu-Zn-SOD, Mn-SOD, and extracellular EC-SOD, and each of them is a product of a distinct gene [82, 83]. Cu-Zn-SOD or SOD 1 (EC 1.15.1.1) is localized in cytosol, Mn-SOD or SOD 2 (EC 1.15.1.1) in mitochondria, and EC-SOD or SOD 3 (EC 1.15.1.1) in extracellular space [84, 85]. Superoxide reacts rapidly with nitric oxide (NO), reducing NO bioactivity and producing the oxidative peroxynitrite radical [86]. SOD, a major defender against superoxide, in the kidneys during the development of murine diabetic nephropathy and downregulation of renal SOD (SOD 1 and SOD 3) may play a key role in the pathogenesis of diabetic nephropathy [87]. Overexpression of SOD or the supplements of antioxidants including SOD mimetics, targeted to overcome oxidative stress, reduce ROS, and increase antioxidant enzymes, has been shown to prevent diabetes mellitus [88].

EC-SOD is found in the extracellular matrix of various tissues including pancreas, skeletal muscle, and blood vessels, and is the major extracellular scavenger of superoxide radicals [89]. The higher level of EC-SOD resulted in a 6-fold increase in the total superoxide dismutase activity of the islets; therefore, superoxide radicals secreted to the extracellular space does not contribute to the *β*-cell destruction [90]. The elevated level of SOD is shown to reduce oxidative stress; decrease mitochondrial release of cytochrome C and apoptosis in neurons; and, in mice, prevent diabetes-induced glomerular injury, thus suggesting a major role of SOD in the regulation of apoptosis [91]. Decline in the level of SOD in diabetic tissue and blood has been reported in many studies [92–94]. Recently Kim (2013) reported that diabetic skin tissues express a relatively small amount of extracellular protein and concluded that extracellular SOD is related to the altered metabolic state in diabetic skin, which elevates ROS production [95]. Study performed by Lucchesi and colleagues [96] to observe the oxidative balance of diabetic rats reported diminished activity of SOD and other antioxidative enzymes in in the liver tissue.

## 5. Antioxidant Potential of Plasma

Antioxidant capacity of plasma is the primary measure and marker to evaluate the status and potential of oxidative stress in the body. Plasma contains many compounds which function against the oxidative stressors in the body thus protecting the cell and cellular biomolecules from being damaged. The combined action of all the antioxidant molecules in the plasma represents the antioxidant capacity of the plasma. Prevalence of oxidative stress is reported in all processes where reduced/depleted plasma antioxidant potential is reported including aging and hypertension [97, 98].

Increased oxidative damage as well as reduction in antioxidant capacity could be related to the complications in patients with type 2 diabetes. The plasma antioxidant level is significantly lower in diabetic subjects with poor glycaemic control than healthy subjects, while patients with good glycaemic control had plasma antioxidative values similar to controls [99, 100]. Catanzaroa et al. (2013) has reported markedly reduced biological antioxidant potential in sciatic nerve homogenates of diabetic animals. Diabetic oxidative stress coexists with a decrease in the antioxidant status, which can further increase the deleterious effects of free radicals [101].

Study conducted by Korkmaz et al. (2013) on 22 diabetic patients to investigate the status of markers of oxidative stress reported a significant reduced level of antioxidant power, measured in terms of ferric reducing antioxidant potential of plasma in diabetic patients. On the basis of result obtained from their study they concluded that the increase in glucose concentrations can lead to tissue damage by increasing oxidative stress [102].

Increased oxidative stress as well as reduction in antioxidant capacity could be related to the complications in patients with diabetes such as oxidative DNA damage and insulin resistance [99]. Due to decrease in antioxidant potential of plasma, complications of diabetes increase which include cardiovascular disease, nerve damage, blindness, and nephropathy. Thus, the increasing incidence of diabetes is a significant health concern beyond the disease itself [103].

## 6. Conclusion

Diabetes is a major source of morbidity, mortality, and economic cost to society. The prevalence of diabetes is rising worldwide due to population growth, aging, urbanisation, and the increase of obesity due to physical inactivity. Oxidative stress plays pivotal role in progression and development of diabetes and its complications. Therapies, consumable or behavioural having capacity to reduces the impact of oxidative stress, may be beneficial to deplete diabetic associates interventions.
